# Contemporary extracorporeal cardiopulmonary resuscitation programs for cardiac arrest

**DOI:** 10.3389/fcvm.2026.1761019

**Published:** 2026-07-08

**Authors:** Samuele Bugo, Alice Bottussi, Jacopo D’Andria Ursoleo, Marta Velia Antonini, Erika Cordella, Myriam Aprile, Samuel I. Garcia, Omar Elmadhoun, Erin D. Wieruszewski, Justyna Swol, Patrick M. Wieruszewski, Fabrizio Monaco

**Affiliations:** 1 Department of Anesthesia and Intensive Care, IRCCS San Raffaele Scientific Institute, Milan, Italy; 2 Department of Medical and Surgical Sciences (DIMEC), University of Bologna, Bologna, Italy; 3 Anesthesia and Intensive Care Unit, Bufalini Hospital-AUSL Della Romagna, Cesena, Italy; 4 Coordination for Transplants’ Center (CRT-ER), IRCCS Azienda Ospedaliero-Università di Bologna, Bologna, Italy; 5 Division of Pulmonary and Critical Care Medicine, Mayo Clinic, Rochester, MN, United States; 6 Department of Emergency Medicine, Mayo Clinic, Rochester, MN, United States; 7 Department of Anesthesiology, Mayo Clinic, Rochester, MN, United States; 8 Department of Pharmacy, Mayo Clinic, Rochester, MN, United States; 9 Department of Respiratory Medicine, Paracelsus Medical University, Nuremberg, Germany; 10 Cardiac Surgery and Extracorporeal Life Support, Department of Cardio-Thoracic Surgery, ECLS Program, Heart & Vascular Centre MUMC+, Cardiovascular Research Institute Maastricht (CARIM), Maastricht, Netherlands; 11 Cardiothoracic and Vascular Anesthesia and Intensive Care, IRCCS Azienda Ospedaliero-Universitaria Sant'Orsola, Bologna, Italy

**Keywords:** cardiac arrest, critical care, ECPR programs, emergency services, extracorporeal cardiopulmonary resuscitation, out-of-hospital cardiac arrest

## Abstract

Cardiac arrest is a leading cause of global mortality, with low survival rates despite advances in resuscitation science. Reported survival ranges from 8% to 34% for out-of-hospital and in-hospital cardiac arrest. Extracorporeal cardiopulmonary resuscitation (ECPR) has emerged as a rescue therapy for patients unresponsive to conventional CPR, leveraging veno-arterial extracorporeal membrane oxygenation (ECMO) to provide artificial circulation, gas exchange, and maintenance of organ perfusion in the absence of spontaneous native circulation. Successful ECPR depends on minimizing time to ECMO initiation and selecting appropriate patients. While several aspects are consistent across countries, ECPR protocols vary in logistics, team composition, cannulation methods, and monitoring, which may influence patient-important outcomes such as survival and neurological recovery. This narrative review explores current global practices in ECPR, and identifies opportunities for standardization while recognizing elements that should remain context specific.

## 1 Introduction

Cardiac arrest remains a significant public health challenge and a leading contributor to global mortality. In Europe, out-of-hospital cardiac arrest (OHCA) is the third most common cause of death (1, 2), with rates of survival to hospital discharge between 8% (3) and 10% (1). In-hospital cardiac arrest (IHCA) occurs at a rate of 1.5 to 2.8 cases per 1,000 hospital admissions, with survival at 30 days or hospital discharge ranging from 15% to 34% (4). Despite advances in resuscitation science, such as early conventional cardiopulmonary resuscitation (cCPR) (5) and the broader deployment of automated external defibrillators (6), the prognosis of patients suffering cardiac arrest remains unacceptably poor.

Extracorporeal cardiopulmonary resuscitation (ECPR) has emerged as a rescue therapy for select patients in whom cCPR fails to restore spontaneous circulation, provided the necessary infrastructure and expertise are available (7–12). By establishing artificial extracorporeal circulation and thereby restoring effective perfusion pressure and systemic oxygen delivery via veno-arterial extracorporeal membrane oxygenation (V-A ECMO), ECPR reduces the duration of the “low-flow” state, the period between initiation of cCPR and return of adequate circulation (13). Major keys to optimizing outcomes include minimizing the interval from cardiac arrest to ECMO initiation and applying clear selection criteria (14). Successful implementation requires coordinated multidisciplinary expertise, rapid team activation, and technical proficiency in cannulation.

**Figure 1:**
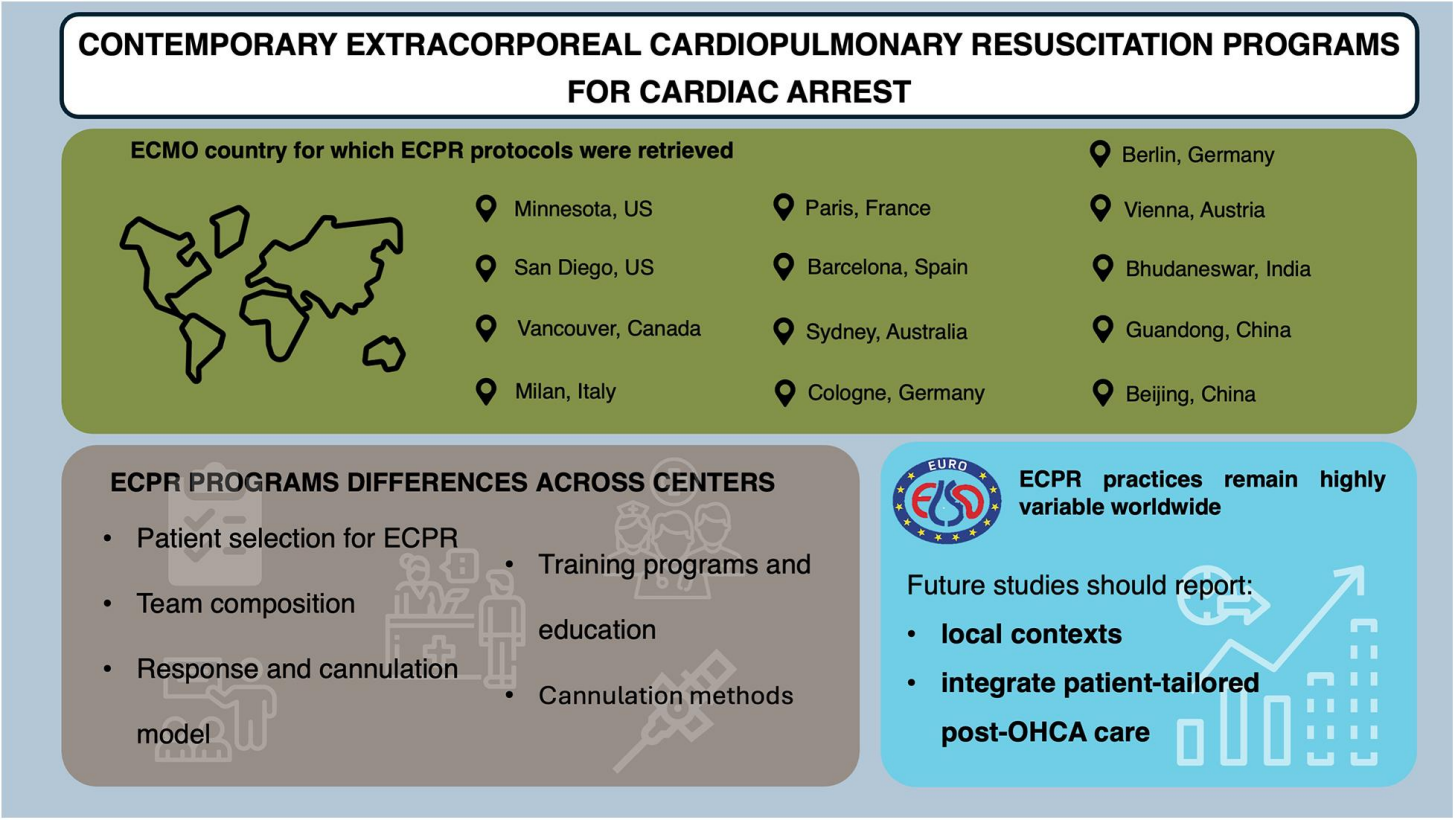
Map illustrating the global distribution of ECMO centers. The green-shaded areas on the map highlight regions with ELSO registered centers (available at: https://www.elso.org), while the boxes indicate the ECMO centers for which ECPR protocols were retrieved and presented in the present review. ECMO, extracorporeal membrane oxygenation; ECPR, extracorporeal cardiopulmonary resuscitation; ELSO, Extracorporeal Life Support Organization.

To date, several ECPR protocols have been developed in expert centers across different countries (Figure 1). Nevertheless, most differ in organizational aspects—such as team composition, cannulation site, size of cannulas, and patient monitoring—that extend beyond the technical elements outlined in the recent 2021 Interim Guideline Consensus Statement of the Extracorporeal Life Support Organization (ELSO) on ECPR in adults, yet may potentially impact outcomes such as survival and neurological recovery (15–17). Furthermore, standardization of protocols may serve to reduce performance bias (18, 19) and clarify factors driving ECPR effectiveness. However, widespread standardization runs the risk of harmful homogeneity due to specific geographic, resource, and logistical variability, and thus must be done so carefully.

**Figure 2:**
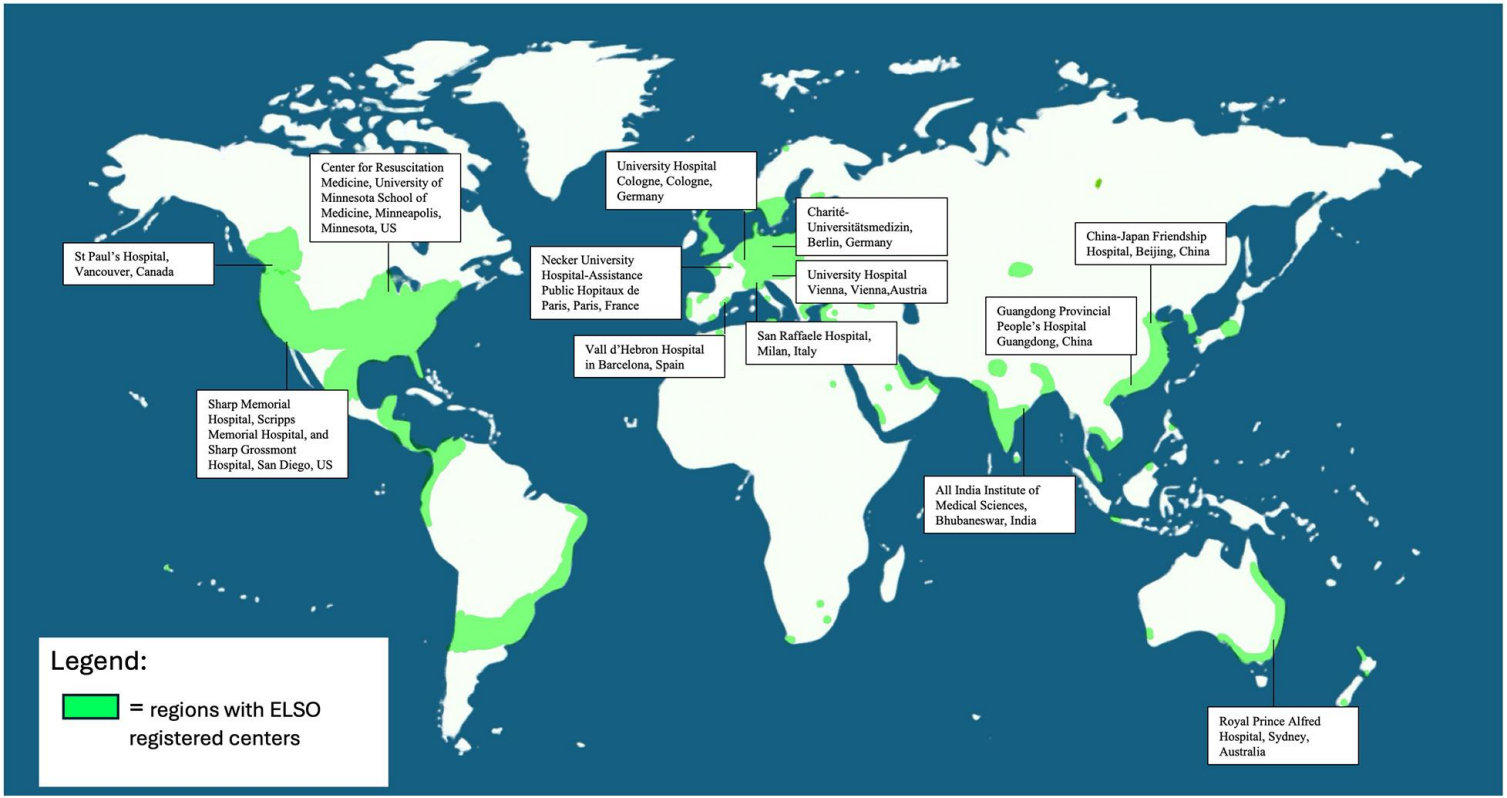
Schematic representation of the participating countries, research methodology, main results and implications. CPR, cardiopulmonary resuscitation; ECPR, extracorporeal cardiopulmonary resuscitation; ELSO, Extracorporeal Life Support Organization.

The objective of this narrative review is to therefore examine the variability among ECPR protocols developed in centers across different countries and identify components that could be standardized globally and those that may need to remain context specific (Figure 2).

## 2 Materials and methods

This review followed the Scale for the Assessment of Narrative Review Articles (SANRA) (20) and applied the SPIDER (Sample, Phenomenon of Interest, Design, Evaluation, and Research type) framework (21) to formulate the research question: what are the characteristics of contemporary ECPR programs, including selection criteria, team composition, and operational logistics? A comprehensive search was conducted by two experienced investigators (SB, AB) across PubMed/MEDLINE, EMBASE, and Google Scholar from inception to August 31, 2025, to identify pertinent literature (22). The search strategy focused on the use of relevant keywords, such as “extracorporeal cardiopulmonary resuscitation”, its related acronym “ECPR”, and “refractory cardiac arrest” in combination with terms including “program”, “strategies”, and “experience”. The Boolean operators “OR” and “AND” were used to enhance search precision. Forward and backward snowballing techniques and consultation with experts in the field were employed to identify additional pertinent literature. EndNote X9 (Clarivate Analytics) was used to identify and remove duplicates. The retrieved studies were subsequently uploaded to Rayyan (Cambridge, MA) and the titles and abstracts were independently assessed for potential inclusion by two investigators (SB, AB) (23). Full-text documents of articles passing the title and abstract review stage were retrieved and independently assessed for final inclusion by both investigators. In case of disagreements during either review stage, a third investigator (JDU) was involved to ensure a mutually agreeable solution and resolve any discrepancies. Only articles published in the English language were eligible. Retrieved studies were qualitatively synthesized and categorized into five domains: i) patient selection for ECPR; ii) response system and cannulation model; iii) team composition; iv) cannulation methods; and v) training programs and education. Core features of the different management strategies were summarized, and heterogeneity in findings was interpreted within the broader context of existing literature.

## 3 Key features of ECPR programs

Implementing an ECPR program requires a structured, multidisciplinary approach that engages a broad spectrum of stakeholders, including hospital leadership, clinical teams, and pre-hospital emergency services. The decision to establish such a program should be based on careful assessment of both its financial implications and anticipated clinical outcomes. Following this preliminary phase, focus can be directed toward team organization, definition of patient selection criteria, development of ECMO-related systems and protocols, and establishment of credentialing, continuous education, and simulation-based training pathways. These programmatic components, while forming the core of ECPR implementation, are inevitably influenced by local infrastructure, resource availability, and the organizational framework of the healthcare system. Consequently, variation across centers and regions is expected, alongside areas of alignment.

### 3.1 Patient selection for ECPR

Identifying patients who are most likely to benefit from ECPR and survive with favorable neurological outcome is challenging, yet crucial. To date, universally accepted inclusion and exclusion criteria for ECPR application are lacking. Nevertheless, the 2021 ELSO Interim Guideline Consensus Statement (17) proposed an example of inclusion criteria for ECPR pathways, which should be tailored to locally available resources and infrastructures (Table 1). Accordingly, three randomized controlled trials (RCTs) comparing ECPR with conventional CPR in OHCA patients reported similar inclusion criteria (Table 2)*.* Specifically, the Advanced REperfusion STrategies for refractory cardiac arrest (ARREST) trial randomized patients with i) age 18–75 years; ii) ventricular fibrillation; iii) estimated transport time to hospital < 30 min; and iv) no return of spontaneous circulation (ROSC) after 3 shocks (24). The Hyperinvasive Approach in Cardiac Arrest (or “Prague OHCA”) trial randomized patients with i) age 18–65 years; ii) arrest from suspected cardiac cause; iii) witnessed collapse; and iv) no ROSC after 5 min of CPR (7). The Early Initiation of Extracorporeal Life Support in Refractory Out-of-Hospital Cardiac Arrest (INCEPTION) trial—the only multicenter RCT—included patients with i) age 18–70 years; ii) ventricular arrhythmia; iii) witnessed collapse with bystander CPR; iv) no ROSC after 15 min (25).

**Table 1:** ECPR inclusion criteria proposed by the ELSO 2021 interim guideline consensus statement on ECPR in adults. ECPR, extracorporeal cardiopulmonary resuscitation; ELSO, Extracorporeal Life Support Organization.

Criteria proposed by the Extracorporeal Life Support Organization (ELSO) in the 2021 Interim Guideline Consensus Statement on Extracorporeal Cardiopulmonary Resuscitation (ECPR) in adults (17)
Age < 70 years
Witnessed cardiac arrest
No-flow interval < 5 min
Initial rhythm of VT, pulseless VT or PEA
Low-flow interval (time from arrest to establishment of ECMO support) < 60 min unless intermittent ROSC, pre-cardiac arrest hypothermia, young age, or signs of life (i.e., gasping, pupillary light reaction, increased level of consciousness) (52) during CPR are present
End-tidal CO_2_ > 10 mmHg during CPR
Intermittent ROSC or recurrent VT
Absence of life-limiting comorbidities (e.g., end-stage heart failure, chronic obstructive pulmonary disease, end-stage kidney disease, liver failure, terminal illness) and ECPR is consistent with patients’ goals of care
Signs of life during CPR
No known aortic valve insufficiency

CPR, cardiopulmonary resuscitation; ECPR, extracorporeal cardiopulmonary resuscitation; PEA, pulseless electrical activity; ROSC, return of spontaneous circulation; VT, ventricular tachycardia.

**Table 2:** ECPR inclusion criteria and main findings of three RCTs comparing ECPR versus conventional CPR in OHCA patients. CPR, cardiopulmonary resuscitation; RCT, randomized controlled trial; ECPR, extracorporeal cardiopulmonary resuscitation; OHCA, out of hospital cardiac arrest.

Trial (reference)	Principal location	No. of patients enrolled	Study setting	Inclusion criteria	Primary endpoint	Key findings
ARREST (24)	USA	30	Single-center	• Age 18–75 years • Ventricular fibrillation • Estimated transport time < 30 min • Absence of ROSC after three shocks	• Hospital survival	• Increased hospital survival in the ECPR group compared to standard of care (43% versus 7%, respectively)
Prague OHCA (7)	Czech Republic	256	Single-center	• Age 18–65 years • Suspected cardiac cause • Witnessed collapse •Absence of ROSC after 5 min of CPR	•Neurologically favorable survival after 180 days	• No difference in neurologically favorable outcome (defined as CPC ≤ 2) at 180 days in the ECPR group compared to standard of care (32% versus 22%, respectively)
INCEPTION (25)	Netherlands	133	Multi-center	• Age 18–70 years • Ventricular arrhythmia • Witnessed collapse with bystander CPR • Absence of ROSC after 15 min	• Neurologically favorable survival after 30 days	• No difference in neurologically favorable outcome (defined as CPC ≤ 2) at 30 days in the ECPR group compared to standard of care (20% versus 16%, respectively)

CPC, cerebral performance category; CPR, cardiopulmonary resuscitation; ECPR, extracorporeal cardiopulmonary resuscitation; ROSC, return of spontaneous circulation.

These criteria have been adopted in a variable fashion worldwide (Table 3). A recent expert consensus on ECPR for OHCA in China recommended the use of ECPR for cases characterized by: i) an ideal no-flow time of < 5 min (extendable to < 10 min); ii) the presence of an initial shockable rhythm, transient ROSC during CPR, shockable rhythms emerging during CPR, or recurrent cardiac arrest episodes; iii) patient age 18 to 70 years—a criterion that may be waived in selected patients with preserved organ function who are likely to exhibit a favorable neurological prognosis; and iv) cannulation for ECPR should begin within 10 to 20 min of refractory cardiac arrest, with ECMO established within 60 min of cardiac arrest (26). Similar criteria were used to establish the Cologne Hospital ECPR program in Germany, which included patients with a no-flow time < 5 min, age ≤ 75 years, and pre-hospital CPR time < 60 min (27). Instead in Berlin, patients were included if: i) cardiac arrest was witnessed; ii) bystander CPR was performed; iii) there was absence of frailty and active malignant disease; iv) the presumed time to ECPR was less than 90 min; v) mechanical compression devices were used; vi) cannulation was technically feasible; and vii) there was no critical bleeding (28). Results of a web-based survey conducted in India identified common exclusion criteria among ECPR programs across the country, including age ≥ 80 years, terminal stage malignancy, pre-existing multiple organ dysfunction, bedridden status or lack of self-independence, severe acute neurological injury, and refractory infections. Most practitioners reported a no-flow time < 5 min and a low-flow time < 30 min for patients selected for ECPR, with the ECMO team typically contacted within 15 min of cardiac arrest refractory to cCPR (29). The regional San Diego County (California, USA) program identified similar exclusion criteria: i) age < 18 or > 70 years; ii) unwitnessed cardiac arrest; iii) non-shockable rhythm or < 2 shocks; iv) absence of mechanical compression devices; v) predicted time from OHCA to ECPR receiving centers > 45 min; vi) ROSC prior to ECPR; and vii) do not resuscitate patients (30). An observational study conducted at a high-volume center in Northern Italy described the inclusion criteria for its ECPR program as follows: i) age 12 to 70 years; ii) witnessed cardiac arrest; iii) no-flow < 12 min; iv) estimated time from cardiac arrest to hospital arrival < 60 min; v) mechanical CPR during transport; vi) end-tidal CO_2_ > 10 mmHg after 20 min of CPR (16). Criteria similar to those proposed by ELSO—including age < 70 years, witnessed cardiac arrest, no-flow time < 5 min, low-flow time < 60 min, cardiac electrical activity at first evaluation, a suspected reversible cause of arrest, and the absence of major comorbidities—were adopted and described in an observational study conducted at Vall d’Hebron Hospital in Barcelona, Spain (15). In Canada, Grunau et al. developed an ECPR program which includes patients with age < 66 years, witnessed arrest, early CPR and one of the following causes of arrest: i) no obvious non-cardiac cause; ii) overdose of cardiac toxin; or iii) hypothermia (31). Lastly, in Vienna, ECPR centers require: i) a witnessed arrest; ii) CPR within 5 min of the arrest; iii) an initial shockable rhythm or pulseless electrical activity with a rate of more than 50 beats per minute; iv) age between 18 and 70 years; v) an estimated center arrival time of less than 40 min; vi) a reversible cause of cardiac arrest; and vii) no underlying condition that would limit intensive care (32).

**Table 3:** ECPR inclusion criteria in different ECMO centers worldwide. ECPR, extracorporeal cardiopulmonary resuscitation; ECMO, extracorporeal membrane oxygenation.

Country	First Author, Year (reference)	ECPR Selection Criteria
China	Wei et al., 2025 (26)	• No-flow of < 5 min (extendable to > 10 min). • Presence of an initial shockable rhythm, transient ROSC during CPR, shockable rhythms emerging during CPR, or recurrent CA episodes. • Age 18–70 years with preserved organ function and likely favorable neurological prognosis. • Cannulation for ECPR might reasonably begin within 10–20 min of refractory CA. • ECMO should be established within 60 min of CA
Germany (Cologne)	Djordjevic et al., 2022 (27)	• No-flow time < 5 min • Age ≤ 75 years • Pre-hospital CPR time < 60 min
Germany (Berlin)	Nee et al., 2020 (28)	• Witnessed cardiac arrest • Bystander CPR • No frailty or active malignant disease • Presumed time to ECPR < 90 min • Use of mechanical compression devices • Technical feasibility of cannulation • No evidence of critical bleeding
India	Gulla et al., 2021 (29)	• ECMO after 15 min of CA refractory to conventional CPR • No-flow time < 5 min • Low-flow time < 30 min
Italy	Scquizzato et al., 2024 (16)	• Age 12–70 years • Witnessed cardiac arrest • No-flow < 12 min • Estimated time from CA to hospital arrival < 60 min • Mechanical CPR during transport • End-tidal CO_2_ > 10 mmHg after 20 min of CPR
Spain	Martìnez-Martìnez et al., 2024 (15)	• Age < 70 years • Witnessed cardiac arrest • No-flow time < 5 min • Low-flow time < 60 min • Presence of cardiac electrical activity at first evaluation • Reversible suspected cause • Absence of major comorbidities
US (San Diego)	Shinar et al., 2025 (30)	• Age 18–70 • Witnessed cardiac arrest • Shockable rhythm or > 2 shocks • Use of mechanical compression devices • Predicted time from OHCA to ECPR receiving centers < 45 min
Canada	Grunau et al., 2017 (31)	• Age < 66 years • Witnessed arrest • Early CPR • One of the following causes of arrest: i) no obvious non-cardiac cause; ii) overdose of cardiac toxin; iii) hypothermia
Austria	Magnet et al., 2025 (32)	• Witnessed arrest • CPR within 5 min of the arrest • Initial shockable rhythm or PEA > 50 • Age 18–70 • Estimated center arrival time <40 min • Reversible cause of cardiac arrest • No underlying condition that would limit intensive care

CA, cardiac arrest; CPR, cardiopulmonary resuscitation; ECMO, extracorporeal membrane oxygenation; ECPR, extracorporeal cardiopulmonary resuscitation; PEA, pulseless electrical activity; ROSC, return of spontaneous circulation.

While practices may vary among centers, and criteria differ, most ECPR programs show a degree of consistency in patient selection. The most common criteria include the presence of refractory cardiac arrest, patient age (upper limit around 70 years, lower limit 18 years), witnessed cardiac arrest, no-flow time of < 5–10 min, low-flow time < 60 min (although with wide variability), and an initial shockable rhythm (33). A retrospective analysis of a multi-regional Australian dataset compared liberal vs. restrictive enrollment criteria from five different ECPR programs across Australian centers (34). By applying different sets of criteria to the ECPR database, the authors noted those that were more restrictive may yield higher survival rates in performed ECPRs, but risk excluding potential patients who do not meet all criteria yet might still have survived (34). Notably, previous studies have shown that, among all criteria, those most closely associated with favorable outcomes include shorter time to ECPR initiation, an initial shockable rhythm, signs of life during CPR, and intermittent ROSC, characteristics that also tend to be associated with favorable outcomes from conventional CPR (16, 33).

### 3.2 Response system and cannulation model

Several logistical models of ECPR for OHCA have been proposed (Table 4). The first and most common model, known as the “transport model,” involves transferring the patient from the site of arrest to an ECPR-capable hospital facility (26). An observational study conducted at Vall d’Hebron Hospital in Barcelona, Spain, which utilizes this model, evaluated outcomes for both IHCA and OHCA. The study reported a median [interquartile range (IQR)] low-flow time of 52 (35–75) minutes, a median cannulation time of 15 (10–19) minutes, a failure rate of 5.6%, and survival rates of 24.1% for OHCA and 33.3% for IHCA (15). Similarly, an observational study conducted in Korea reported a median cannulation time of 25 (20–31) minutes and a survival rate of 18% using the transport model (35). Another randomized trial conducted in Prague, Czech Republic, failed to demonstrate a significant difference in survival or neurological outcomes between an early transport invasive strategy and standard care (7). Specifically, the invasive approach involved immediate intra-arrest transfer of patients to a specialized cardiac center for consideration of ECPR, whereas standard of care consisted of continued on-scene advanced cardiac life support, including chest compressions, drugs, defibrillation, vasoactive medications, and other guideline-directed resuscitative interventions until ROSC was achieved (7). While the trial found no significant difference in survival with neurologically favourable outcomes [31.5% in the invasive strategy group vs. 22% in the standard strategy group at 180 days, OR 1.63 (95% CI, 0.93 to 2.83)], the trial was terminated by the data and safety monitoring board, having met the prespecified criteria for futility.

**Table 4:** Pre-hospital extracorporeal cardiopulmonary resuscitation models.

	Transport model	Convergence model	On-site model
Description	The patient is transferred from the site of cardiac arrest to a hospital facility capable of providing ECMO implantation	The patient is transferred to the nearest medical facility by a mobile ECMO team for the initiation of ECPR. Following ECMO implantation, the patient is subsequently transferred to a catheterization laboratory or an ECMO center for further management	A trained ECPR team is dispatched directly to the cardiac arrest site to evaluate the patient and, when appropriate, initiate ECPR
Cannulation site	ECPR hospital facility	The nearest medical facility	On-site
Example programs	Vall D’Hebron Hospital, Barcelona (Spain) IRCCS San Raffaele Hospital, Milan (Italy) University Hospital Cologne, Cologne (Germany) Sharp Memorial Hospital, Scripps Memorial Hospital, and Sharp Grossmont Hospital, San Diego (USA) St Paul's Hospital, Vancouver (Canada) Charité-Universitätsmedizin, Berlin (Germany) University Hospital Vienna, Vienna (Austria)	Center for Resuscitation Medicine, University of Minnesota (USA)	Royal Prince Alfred Hospital, Sydney (Australia) Necker University Hospital, Paris (France)

ECMO, extracorporeal membrane oxygenation; ECPR, extracorporeal pulmonary resuscitation.

The second proposed model, referred to as the “convergence model,” involves transporting the patient to the nearest medical facility, where ECPR is initiated by a specialist mobile team dispatched from a referral center, followed by the transfer to a facility equipped with a catheterization laboratory or ECMO center for further management (26). An observational study conducted by the Center for Resuscitation at the University of Minnesota in Minneapolis, USA assessed the effectiveness of a mobile ECMO team able to report to three designated hospitals to cannulate. The study reported a mean ± standard deviation (SD) time of 46.9 ± 12.3 min between the initial emergency call and patient arrival at the emergency department (ED). The mean time from ECMO team activation to arrival to the designated ED was 14.9 ± 5.7 min, with 71% of cases presenting a response time within 15 min (36). Furthermore, the mean time from patient arrival at the nearest facility to ECMO initiation was 14.4 ± 6.1 min, with 63% of cases undergoing ECMO initiation within 15 min (36). Successful cannulation and transportation were achieved without adverse events in all cases, resulting in a 43% functionally favorable survival rate (36). From observational studies, the survival rate of the convergence model appears to be higher compared to the transport model. While a potential explanation for this observation could lie in the slightly lower low-flow times reported in the convergence model, more careful evaluation of such data is warranted, given the lack of comparative data available in the published literature. Therefore, further research is warranted to fully characterize the risks and benefits of this approach, given its potential utility in large metropolitan areas with few ECMO centers and several smaller, interspersed hospitals.

The third model, referred to as the “on-site model”, involves deploying a trained ECPR team directly to the site of cardiac arrest to evaluate the patient and, if appropriate, to promptly initiate ECMO (26). A pilot feasibility study conducted in Sydney, Australia, reported a mean cannulation time of 14 min, a mean total low-flow time of 39 min, and a survival rate of 25% using the on-site model (37). Additionally, Song et al. compared the three models in Australia, considering factors such as the number of resident population members able to access ECPR and be initiated on ECMO within 1 h of arrest, the population-weighted average survival probability assuming a 1-hour eligibility cutoff, and the number of expected survivors based on the area-wide incidence of ECPR-eligible OHCA cases. Their analysis found that the on-site cannulation model resulted in an improved survival rate (38). Case reports of successful ECMO cannulation in unusual locations, such as the Louvre Museum in Paris, France, have also been published under this model (39).

Ultimately, the selection of the most appropriate model should be based on both logistical and operator-dependent factors and evaluated on a case-by-case basis within the context of local healthcare systems organization and resource availability.

### 3.3 Team composition

The importance of a multidisciplinary ECMO team has been well recognized and discussed in the available literature (40–42). The 2021 ELSO Interim Guideline Consensus Statement on ECPR in adults emphasizes teamwork with clearly defined roles and highly specialized healthcare providers; however, it does not specify the precise and most appropriate composition of the team (17). Rather, it outlines the functions of different subgroups (e.g., pre-hospital team, cannulation team, cardiology team) without clarifying which medical professionals should assume these roles or how many individuals should be assigned to each (17). As a result, team composition largely depends on the expertise and financial resources of individual ECMO centers, which may influence team efficiency and patient outcomes.

Bertini et al. proposed forming an ECPR team based on the transport model, recommending the inclusion of specialists from the fields of emergency medicine, intensive care, cardiothoracic, vascular, and general surgery, as well as cardiology, anesthesiology, radiology, and palliative care (43). The ECPR location should be strategically selected and may include the ED, catheterization laboratory, hybrid operating room, or intensive care unit (ICU) (43). An observational study conducted at a high-volume center in Northern Italy reported an ECMO team composed of two cardiothoracic intensivists, a cardiologist, two ICU nurses, and a cardiovascular perfusionist, guided remotely by a senior ICU physician located in the receiving cardiothoracic ICU (16). Similarly, the Berlin ECPR program involves a multidisciplinary team in an ICU setting (28). The team is composed of a team leader, an attending physician as the pump operator and supervisor, an intensivist responsible for cannulation, three specialized nurses, and a physician-in-training (28). In Canada, the ED involves an emergency team composed of two emergency physicians, four nurses, and one respiratory therapist, and an ECMO team composed of a cardiovascular surgeon assisted by an emergency physician (31). Additional personnel involved include cardiac anesthesiologists, cardiac perfusionists, ICU staff, the cardiac surgery ICU nurse leader, and the hospital clinical coordinator (31). In Vienna, the team includes a cardiac surgeon or an ED physician responsible for ECMO cannulation, while ED personnel perform a clinical assessment and use transesophageal echocardiography to identify reversible causes of cardiac arrest (32). Instead, in San Diego County, ECPR is performed by a team of emergency physicians, including a leader and two cannulating physicians (30).

In China, a recent Expert Consensus on Extracorporeal Cardiopulmonary Resuscitation for Out-of-Hospital Cardiac Arrest recommended that specialized pre-hospital teams—organized under the Mobile ICU on-site model—include emergency or intensive care physicians, pre-hospital nurses, surgical specialists, and other relevant personnel (26). In a multicenter study, Hutin et al. proposed an example of a Mobile ICU team consisting of: i) a resuscitation team leader (physician or advanced paramedic), responsible for supervising advanced cardiac life support interventions, establishing airway and intravenous access, obtaining medical history, and liaising with the ECPR team; ii) a resuscitation nurse/paramedic, tasked with ventilatory management, drug administration, and maintaining advanced cardiac life support flow and timekeeping; iii) a paramedic/emergency medical services technician/second nurse responsible for scene management and operation of the mechanical CPR device; iv) an ECPR physician, who collaborates with the team leader in patient selection, performs cannulation, and supervises implantation; v) an ECPR nurse, who pre-assembles the extracorporeal circuit and manages post-pump critical care; and vi) an ECPR paramedic/second nurse, who manages ECPR equipment and assists the ECPR physician (44).

Similarly, the Center for Resuscitation at the University of Minnesota in Minneapolis, USA, following the convergence model, established a mobile ECMO cannulation team composed of a senior physician experienced in cannulation, specialists in interventional cardiology, emergency medicine, and critical care, as well as a sterile and a non-sterile assistant (36). The team remains at the patient's bedside from the ED cannulation through transport to the centralized ECMO ICU, providing continuous resuscitation and optimization of ECMO management as needed (36).

### 3.4 Cannulation methods

The 2021 ELSO Interim Guideline Consensus Statement on ECPR in adults outlines two main approaches: a percutaneous technique, typically performed using the modified Seldinger method, and a hybrid technique involving surgical exposure of the femoral vessels (i.e., “cutdown approach”) (17). Evidence suggests that the surgical approach is inferior to the percutaneous technique and is therefore generally intended as a backup strategy only (45). Consequently, most centers adopt the percutaneous method as their primary approach. However, the guidelines provide limited detail regarding cannula size, anatomical site, or the optimal execution of the percutaneous technique.

According to the ELSO Interim Guideline, the optimal percutaneous approach, without anatomical site preference, should include i) the use of an arterial cannula sized 15–17 Fr and a venous cannula sized 19–25 Fr; ii) ultrasound visualization of the vessels; iii) confirmation of venous and arterial guidewire placement using transthoracic or transesophageal echocardiography, or fluoroscopy when available; and iv) flushing of the first cannula with heparinized saline solution (17). The greatest variability among centers lies in the choice of cannula size, the preferred anatomical cannulation site, the method used to confirm guidewire position, and use of anticoagulation.

The ECPR program at Vall d’Hebron Hospital in Barcelona, Spain, employs an ultrasound-guided percutaneous technique with a right-sided femoro-femoral approach. The standard configuration consists of a 23–25 Fr, 55 cm long multistage drainage cannula, and a 15–17 Fr, 15 cm long return cannula. Guidewire placement is typically confirmed using transthoracic echocardiography from the subcostal view except in patients located in the catheterization laboratory, where fluoroscopy guidance is available and usually provided by interventional cardiologists (15). Similarly in Berlin, cannulation of the femoral vein and artery is aided by ultrasound with a 15–17 Fr arterial cannula and 23 Fr venous cannula (28).

A high-volume center in Northern Italy reported performing V-A ECMO cannulation with a percutaneous femoro-femoral approach—without specifying whether ipsilateral or contralateral—under transesophageal echocardiographic guidance, using a 29 Fr venous drainage cannula and a 15–17 Fr arterial cannula (16). In Canada, Grunau et al. reported a femoro-femoral approach, using a 17 Fr cannula arterial cannula and a 23 Fr venous cannula (31). In China, a recent expert consensus on ECPR for OHCA recommends percutaneous vascular access for pre-hospital ECMO cannulation, with guidewire insertion confirmed by ultrasound. The suggested cannula sizes are 15–17 Fr for the femoral artery and 19–25 Fr for the femoral vein (26). Cannula size ultimately depends on patient anatomy, and the femoro-femoral approach is generally preferred due its ease of access in adults (46). Furthermore, several studies have demonstrated the superiority of transesophageal over transthoracic echocardiography for ECMO cannula positioning, highlighting the advantages of transesophageal echocardiography and supporting its preferential use when available (47, 48).

### 3.5 Training programs and education

The importance of team expertise has been emphasized in several studies, which demonstrated that centers with greater experience and higher case volumes achieved improved patient survival rates—particularly in patients with non-shockable rhythms—as well as better neurological outcomes (32, 49, 50). In line with these findings, the 2021 Interim ELSO Guideline Consensus Statement on ECPR in adults identifies training as the cornerstone of program development, essential for delivering safe and standardized care (17). This principle is consistently reflected across different ECPR programs worldwide, all of which emphasize structured training as a prerequisite for successful implementation. In practice, multidisciplinary team training should commence once consensus on key operational aspects has been established and preliminary system designs are in place. Early educational efforts may include lectures, reference materials, and instructional videos introducing ECMO systems to the relevant stakeholder groups. Feedback from these sessions is critical for refining program design and ensuring its feasibility in clinical settings. Nevertheless, didactic activities alone are insufficient. To ensure readiness for real-world application, training must incorporate regular multidisciplinary *in-situ* simulations, encompassing the full spectrum of ECPR activities such as advanced cardiac life support, ECMO cannulation, advanced imaging, transport, and complication troubleshooting (31, 51). Additionally, insufficient procedural exposure and limited team experience may contribute to the neutral outcomes observed in several trials comparing conventional and extracorporeal CPR. For example, the INCEPTION trial, which reported no significant difference in survival with favorable neurological outcome or in serious adverse events rate between patients treated with ECPR and those receiving conventional CPR, enrolled 136 patients across 10 centers, most of which enrolled only a few patients during the four-year study period (25). Nonetheless, a study from Japan reported conflicting evidence about the potential impact of greater ECPR experience and outcome (49).

## 4 Discussion

ECPR is increasingly utilized for in-hospital and out-of-hospital cardiac arrest that is refractory to cCPR. Patient outcomes tend to be heavily influenced by trial design, selection bias, and specific infrastructure and practices of the program performing cannulation. While guidelines have been developed for ECPR in adults, the evidence base delineating ideal practices remains limited. The amount of published evidence regarding ECPR programs is disproportionate from the total number of centers performing ECMO that are registered with ELSO worldwide. Additional research is needed to further identify the most appropriate patient selection criteria, the optimal model for initial deployment and management of ECMO, and specific infrastructure and resources necessary to operate a successful ECPR program.

## 5 Study limitations

The present study has limitations. First, the narrative nature of this review and the absence of quantitative synthesis limit the strength of the inferences and further constrain the ability to provide a comprehensive overview of ECPR practices. Additionally, the scarcity of literature on the topic, along with the potential risks of publication and confirmation bias, warrants caution as many more ECMO centers are likely performing ECPR beyond those identified in this review. Furthermore, the paucity of evidence from low- and middle-income countries and other resource-constrained settings restricts the generalizability of our conclusions. Lastly, the restriction to articles published in English may have resulted in the omission of relevant regional evidence.

## 6 Conclusions and future perspectives

Although the ELSO 2021 Interim Guideline Consensus Statement on ECPR has been widely adopted, meaningful local heterogeneity persists in transport models, team composition, and both in-hospital and pre-hospital management of ECPR programs. Much of this variability reflects largely nonmodifiable factors related to logistics, healthcare system organization, and emergency services configuration. Consequently, comprehensive global standardization of care pathways is unlikely to be feasible. These contextual factors should therefore be explicitly reported in scientific papers and clinical trials and considered when comparing and interpreting studies. Post-cardiac arrest pathways also play a major role in patient outcomes; yet, despite existing guidance, they remain strongly shaped by patient-specific characteristics and preferences in an era of increasingly individualized medicine. Accordingly, post-OHCA management strategies and their adaptations should be clearly described and incorporated into future research to enable fair and transparent comparisons across studies.
